# Early pH Changes in Musculoskeletal Tissues upon Injury—Aerobic Catabolic Pathway Activity Linked to Inter-Individual Differences in Local pH

**DOI:** 10.3390/ijms21072513

**Published:** 2020-04-04

**Authors:** Julia C. Berkmann, Aaron X. Herrera Martin, Agnes Ellinghaus, Claudia Schlundt, Hanna Schell, Evi Lippens, Georg N. Duda, Serafeim Tsitsilonis, Katharina Schmidt-Bleek

**Affiliations:** 1 Julius Wolff Institut, Charité–Universitätsmedizin Berlin, 13353 Berlin, Germany; julia-catherine.berkmann@charite.de (J.C.B.); aaron.herrera@charite.de (A.X.H.M.); claudia.schlundt@charite.de (C.S.); hanna.schell@web.de (H.S.); evi.mm.lippens@gmail.com (E.L.); georg.duda@charite.de (G.N.D.); serafeim.tsitsilonis@charite.de (S.T.); 2 Berlin-Brandenburg School for Regenerative Therapies, Charité–Universitätsmedizin Berlin, 13353 Berlin, Germany; 3 BIH Center for Regenerative Therapies, Charité–Universitätsmedizin Berlin, Berlin 10178, Germany; agnes.ellinghaus@charite.de; 4 Center for Musculoskeletal Surgery, Charité–Universitätsmedizin Berlin, 13357 Berlin, Germany

**Keywords:** pH change, musculoskeletal system, bone healing, muscle injury, initial healing phase, pH-triggered drug release

## Abstract

Local pH is stated to acidify after bone fracture. However, the time course and degree of acidification remain unknown. Whether the acidification pattern within a fracture hematoma is applicable to adjacent muscle hematoma or is exclusive to this regenerative tissue has not been studied to date. Thus, in this study, we aimed to unravel the extent and pattern of acidification in vivo during the early phase post musculoskeletal injury. Local pH changes after fracture and muscle trauma were measured simultaneously in two pre-clinical animal models (sheep/rats) immediately after and up to 48 h post injury. The rat fracture hematoma was further analyzed histologically and metabolomically. In vivo pH measurements in bone and muscle hematoma revealed a local acidification in both animal models, yielding mean pH values in rats of 6.69 and 6.89, with pronounced intra- and inter-individual differences. The metabolomic analysis of the hematomas indicated a link between reduction in tricarboxylic acid cycle activity and pH, thus, metabolic activity within the injured tissues could be causative for the different pH values. The significant acidification within the early musculoskeletal hematoma could enable the employment of the pH for novel, sought-after treatments that allow for spatially and temporally controlled drug release.

## 1. Introduction

The pH is tightly regulated in the living organism, with values ranging from 7.35 to 7.45. There are only a few situations known in which a deviation from the physiological pH is not associated with sickness—for example, the acidification of muscle tissue during high muscle activity [1,2]. Disease-associated pH deviations occur under conditions in which oxygen cannot be properly transported into the tissues, e.g., after injuries due to a rupture of blood vessels or ischemic disorders [2], which induces local hypoxia [3,4] along with tissue damage. Another reason for perturbation of homeostatic pH is described by the Warburg effect [5] in cancer cells, which favor aerobic glycolysis over oxidative phosphorylation [3], resulting in a decrease in external pH, especially in hypoxic regions of the tumor [6]. All the above examples share a common underlying principle: a change in metabolic processes towards anaerobic lactate fermentation as the main pathway for energy generation by glucose catabolism. This indicates that metabolic activity can alter the local environment, such as when acidic metabolites are transported out of the cell and thereby acidify the microenvironment [7]. Since significant deviations from physiological pH are strongly associated with the disease state of the organism, the pH could be considered a marker for certain diseases. In wound healing, the pH value is suggested as a parameter for both diagnostic and theranostic purposes [8]. Moreover, recent advances in the field of stimuli-responsive drug delivery [9,10] allow for utilization of the pH as an endogenous trigger for drug release in wound healing [11] and solid tumors [9,12,13].

It is well known that the pH is a parameter of utmost importance for bone homeostasis and the skeleton exerts a homeostatic role in buffering excessive acids [14,15,16]. Osteoclasts can be activated by acidic pH, with local acidification in resorption pits being one of the mechanisms of action for bone catabolic processes [17,18]. Osteoblasts, on the contrary, are sensitive to acidosis, which can decrease or even inhibit matrix mineralization [18,19]. Moreover, it is widely accepted that bone fracture evokes a local acidification of the fracture environment [20,21]. However, the timing and the extent of acidification remain largely unknown. Street et al. studied pH changes upon injury by comparing the pH values of supernatants of human fracture hematomas with hemolyzed control plasma [22]. No deviation from physiological pH was observed in either control plasma samples or supernatants collected from hematomas obtained approximately 12 h post fracture. However, the sample preparation method included centrifugation, freezing and thawing [22], a protocol that might have affected the pH. In another study, the relationship between mineralization and pH value was investigated in a rat osteotomy model [23]. The pH of the bone repair tissue was lower than the pH of control serum during the first week after osteotomy with a pH of 7.2 as the lowest value measured at two days post fracture followed by a continuous increase. The finding of an initial pH drop post osteotomy is in line with a number of studies summarized by Newman et al. [24]. Comparing the mean minimum pH values and the recording time points used in these studies, which involved various species from rodents to dogs to humans, the pH values measured within the first 8 days post fracture ranged from 4.2 to 7.19 [24]. Thus, the data remains inconclusive, as there is wide variation in the recorded values between the studies. This may be due to the individual study design and measurement time points, especially for the early healing time points, where data is scarce. Therefore, no reliable statement can be made about the pH of the fracture hematoma.

In this study, we aimed to investigate the changes and dynamics in pH in the musculoskeletal system after injury to define target pH values for novel pH-dependent treatment approaches. To understand how the local pH is affected at early stages of the bone healing cascade and whether the effect on pH due to bone fracture can be compared to other injuries of the musculoskeletal system, a sheep and a rat model were utilized to monitor immediate to early deviations in pH values in vivo up to 48 h after injury. To the best of our knowledge, this is the first study conducting parallel pH measurements in hematomas originating from the injury of two musculoskeletal tissues within the same animal, which therefore excludes issues of animal-specific differences that could affect this comparison. We observed acidification in both fracture and muscle hematomas without clear tissue-dependent effects. At all time points, and in both hematomas, high inter-individual differences were detected. Focusing on the fracture hematoma, we aimed to unravel the cause of this inter-individual variability by studying cellular density and aerobic catabolic activity. While no correlation of cellular density and pH was detected, a link between tricarboxylic acid cycle and local pH could be demonstrated. In light of a continuous demand for novel, pharmacological interventions to treat cases of musculoskeletal impaired healing after trauma, providing actual in vivo local pH values after injury could be of high relevance for the development of stimuli-responsive drug release systems.

## 2. Results

Based on the aim of this study to provide target values for the development of pH-sensitive biomaterials that can be applied in the context of bone regeneration, we focused on early phases post injury and on the hematomas formed after bone or muscle trauma. Bone and muscle are highly vascularized key tissues of the musculoskeletal system. In the clinic, patients usually present with a mixed muscular and skeletal trauma. However, in order to distinguish between hematomas arising from a trauma to either bone or muscle, standardized, isolated defects were chosen. Moreover, in previous studies comparing bone fracture and soft tissue hematoma, we unraveled distinct differences in terms of cellular composition, pro-/anti-inflammatory cytokine secretion and gene expression [25,26]. Therefore, we investigated whether all the above differences are accompanied by variations in pH changes as well.

### 2.1. Sheep Study: pH Drop upon Hypoxia Following Injury

**Figure 1:**
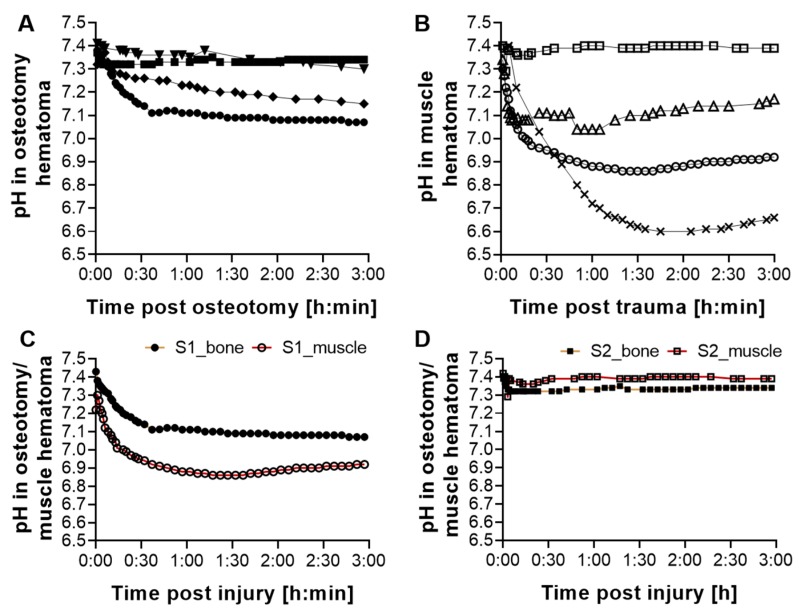
Local acidification in emerging hematomas after osteotomy and muscle trauma. Measurement of pH values over the indicated time in a proximal tibia shaft (**A**) and muscle injury in the Vastus lateralis (**B**) that corresponded in size to the tibia bone injury. Each line in the graph represents the development in local pH for one animal. While a drop in pH was detected in both bone and muscle hematoma for most animals, the inter-individual variations were high. The range of pH variations was broader after muscle trauma. Parallel pH measurement of bone and muscle hematoma for two animals (**C**,**D**) revealed intra-individual variations in addition to the values measured inter-individually. Each line in the graph represents the development in local pH separately for the osteotomy (yellow line) and muscle (red line) hematoma within the same animal (S1 or S2).

For the investigation of changes in the local pH within the hematoma, a pilot in vivo pH measurement in sheep was initiated directly after injury, thereby allowing for the monitoring of changes in the very early phases of healing. This measurement was integrated in another study, thereby following the 3R principles (replace, reduce, refine) [27]. The pH within the osteotomy hematoma (*n* = 4) and the muscle trauma hematoma (*n* = 4) was tracked over three hours (Figure 1A, B). A pH drop was detected in the osteotomy hematoma in the majority of animals (Figure 1A), albeit with pronounced inter-individual variations. After 3 h, the mean pH of all animals was 7.21 ± 0.13. Except for one animal, this local acidification was also detected in muscle hematomas (Figure 1B) with stronger pH deviations and a mean pH of 7.04 ± 0.3.

In order to investigate whether the inter-individual variations would be similar in both muscle and osteotomy hematoma, a parallel pH measurement was conducted in two animals (Figure 1C,D). Interestingly, in addition to the inter-individual variations, intra-individual differences were also observed. While in one animal, the pH in the osteotomy hematoma was lower compared to the muscle pH, it was the opposite in the second animal. Hence, these findings indicate a general acidification of the forming hematomas without clear tissue-dependent effects. Due to the embedding of this study in an ongoing in vivo study, in line with the 3R principles, the number of animals and access to defect sites were restricted, and, furthermore, the time span for measurement was limited to the very early phases of musculoskeletal healing.

### 2.2. Gene Expression Analysis in Sheep Fracture Hematoma

**Figure 2:**
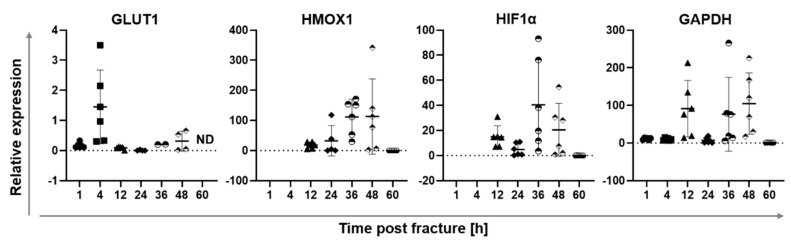
Gene expression in osteotomy hematoma tissue at 1, 4, 12, 24, 36 and 48 h post osteotomy. Samples were generated in an earlier study and gene expression of selected targets of interest was analyzed. *HIF-1α*, *GLUT1* and *HMOX1* have been previously published (adapted from [25]). *HIF-1α* and *HMOX1* expression was analyzed starting from 12 h post fracture. Results are depicted as scatter plots, each point represents the mean gene expression detected in the osteotomy hematoma for one animal. All samples were measured in triplicate. For all animals per time point (*n* = 6) the mean gene expression ± SD is shown. Different signs (circle, square, triangle and rhombus) indicate the different time points. ND: non-detectable expression.

In an earlier study in sheep, the fracture hematoma of a stable fixed osteotomy of the tibia at 1, 4, 12, 24, 36, 48 and 60 h was harvested in order to conduct gene expression analysis [25]. The aim was to understand cellular reactions with respect to changes in the local environment. If the cellular processes were still ongoing, then the cells should express genes allowing for the transport of glucose into the cell due to increased energy demands. In conditions of oxygen deprivation, glycolysis and subsequent lactate fermentation generate less energy/ATP per mol glucose than aerobic respiration, therefore, glucose transporter genes must be upregulated to meet energy demands. Accordingly, the glucose transporter 1 (*GLUT1*) was analyzed to determine enhanced glucose uptake. In addition, hypoxia-inducible factor-1α (*HIF-1α*), a gene upregulated during hypoxia, heme oxygenase-1 (*HMOX1*), a pro-angiogenic, anti-inflammatory and cytoprotective protein [25,28] that can be induced by injury, and glycerinaldehyde-3-phosphate dehydrogenase (*GAPDH*), a classically used house-keeping gene and glycolytic enzyme, were also analyzed (Figure 2). Increased *GLUT1* expression at 4 h post osteotomy indicates that cells try to enhance the import of glucose post injury. *HMOX1* increased in the hematoma with the mounting hypoxic stress, peaking at 36 h post fracture. Similarly, *HIF-1α* showed the highest induction at 36 h post osteotomy, suggesting that the tissue is indeed hypoxic. *GAPDH* has been shown to be regulated by hypoxia in different cell types [29,30]. In line with the literature, *GAPDH*, showing a first peak at 12 h after vessel disruption indicating a shortage of oxygen, followed the pattern of *HIF-1α* expression in this study as well.

This data indicates that the genetic response to developing hypoxia is not immediate, as indicated by the lack of upregulation of *GLUT1* and *GAPDH* genes at 1 h post injury, but develops over time. It is initiated by an upregulation of *GLUT1* expression at 4 h, followed by an increased expression of hypoxia-related and -regulated genes. Combining these insights with the mild decrease in local pH of the fracture hematoma measured in sheep in the pilot in vivo study up to 3 h post fracture (Figure 1) suggests that hypoxic effects and, thus, hypoxia-induced pH acidification could be more pronounced at later time points post fracture. Therefore, another animal study was performed to analyze longer time periods after injury in order to test the hypothesis that lower pH values due to oxygen shortage would emerge with more time.

### 2.3. Rat study: pH Drop during Hematoma Maturation Following Injury

**Figure 3:**
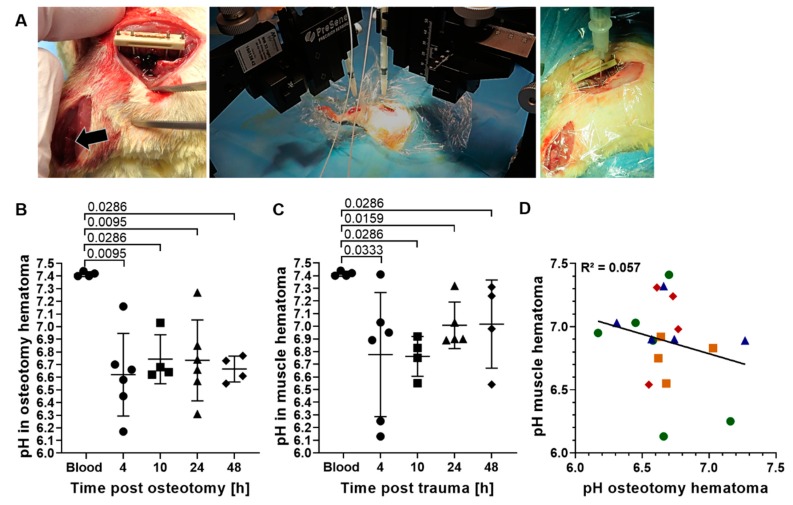
In vivo pH measurement at 4, 10, 24 and 48 h post osteotomy and muscle trauma. (**A**) Surgical approach and in vivo pH measurement. From left to right: muscle trauma and Musculus soleus (arrow) and osteotomy with a 5 mm gap size and an external fixation (RISystems); simultaneous and continuous pH measurement within muscle and osteotomy hematoma using optical microsensors (PreSens Precision Sensing) with precise positioning via micromanipulators; close-up image of placement of the pH sensor in the osteotomy hematoma. (**B**) Scatter plot of individual mean pH of the osteotomy hematoma per time point. (**C**) Scatter plot of individual mean pH of the hematoma formed after muscle trauma per time point. For (**B**) and (**C**): Each value depicted (point per time point) represents the measurement of the respective hematoma for one animal. Mean ± SD is shown for at least four animals per time point. Circles: 4 h, squares: 10 h, triangle: 24 h and rhombus: 48 h time point post injury. Two-way Mann–Whitney U test was performed, comparing the measurement time points to control (blood). (**D**): Local pH in osteotomy hematoma plotted against local pH in muscle hematoma. Linear regression line is depicted for better visualization. Both parameters (*n*= 19) passed the D’Agostino–Pearson normality test (α= 0.05, QQ plot depicted in Supplementary Figure S2A). Hence, Pearson correlation was calculated, yielding an R^2^ value of 0.057. Green circles: 4 h, yellow squares: 10 h, blue triangles: 24 h, and red rhombus: 48 h.

For the analysis of pH development over a longer time period, an in vivo pH measurement in the osteotomy gap of rats at 4, 10, 24 and 48 h post osteotomy was initiated (Figure 3A), which includes the phases of hematoma formation, pro-inflammation and initial anti-inflammatory reaction of the bone healing process with the latter initiating revascularization [31]. Revascularization could be detected in the rat osteotomy hematoma at three days post osteotomy in another study [32]. Hence, with our experimental time interval, we aimed to cover the entire hypoxic phase.

In a first test, the physiological pH in freshly collected blood from rats (*n* = 4) was confirmed to be in the same range as the physiological pH in human blood, with a mean of 7.41 ± 0.02, and was used as comparator for the pH measured in the hematomas.

The in vivo measurement of the osteotomy hematoma at all investigation time points revealed a significant decrease in pH, with mean pH values ranging from 6.62 ± 0.33 to 6.74 ± 0.19 (Figure 3B). The individual pH variations per animal over the measurement period were negligible with a mean SD of 0.02 (Supplementary Figure S1B). However, the inter-individual differences at each time point were substantial, as seen by the rather large variation of the data for the analyzed time points. Similar to the osteotomy hematoma, a significant drop from physiological pH as well as pronounced inter-individual differences were observed within the muscle hematoma at all time points (Figure 3C). The mean pH of muscle hematoma per time point was slightly higher compared to the osteotomy hematoma, ranging from 6.76 ± 0.16 to 7.02 ± 0.35. Moreover, the mean pH per time point was found to increase only for the muscle hematomas between the 10 and 24 h time point from 6.8 to 7.0, while the mean pH in bone hematoma remained constant over the testing period (Figure 3B,C).

The high inter-individual differences seen in the hematomas of both musculoskeletal tissues raised the question whether the extent of acidification in both hematomas would be similar or whether either the bone or muscle hematoma would be consistently more acidic within each individual animal. Since the pH in both hematomas was measured in parallel in the same animal (Figure 3A), the difference between pH values of both tissues could be calculated individually (Table 1). In case of a general pattern of higher acidification of one hematoma group compared to the other, the pH could be postulated to be dependent on the tissue that the hematoma originated from. In line with our findings in sheep (Figure 1C,D), no clear pattern in hematoma acidification was observed over the entire testing period, meaning that for some animals, the osteotomy hematoma was more acidic than the muscle hematoma, while it was the reverse for others (Table 1). Despite the lack of a constant pattern for all time points, at later time points (24 and 48 h), the majority of bone hematomas were found to be more acidic than the muscle hematoma. This finding is supported by a linear regression analysis of the local pH within both hematomas, with each point representing an individual animal (Figure 3D). Here, the calculation of Pearson correlation yielded an R^2^ value of 0.057, indicating that the local pH in osteotomy and muscle hematoma are not correlated.

Similar to the in vivo pH measurement in sheep, the in vivo pH measurement in rats confirmed the acidification within both hematomas, but with a stronger degree of acidification in the rat study. Moreover, in both studies, no clear tissue-dependent effects of pH acidification pattern for bone and muscle hematomas could be detected (Figure 1C,D, Figure 3D, Table 1) and pronounced inter-individual differences within each hematoma group were observed.

### 2.4. Factors Influencing the Variability in Local pH

#### 2.4.1. The Cellularity of the Hematoma Does Not Correlate with Local pH

**Figure 4:**
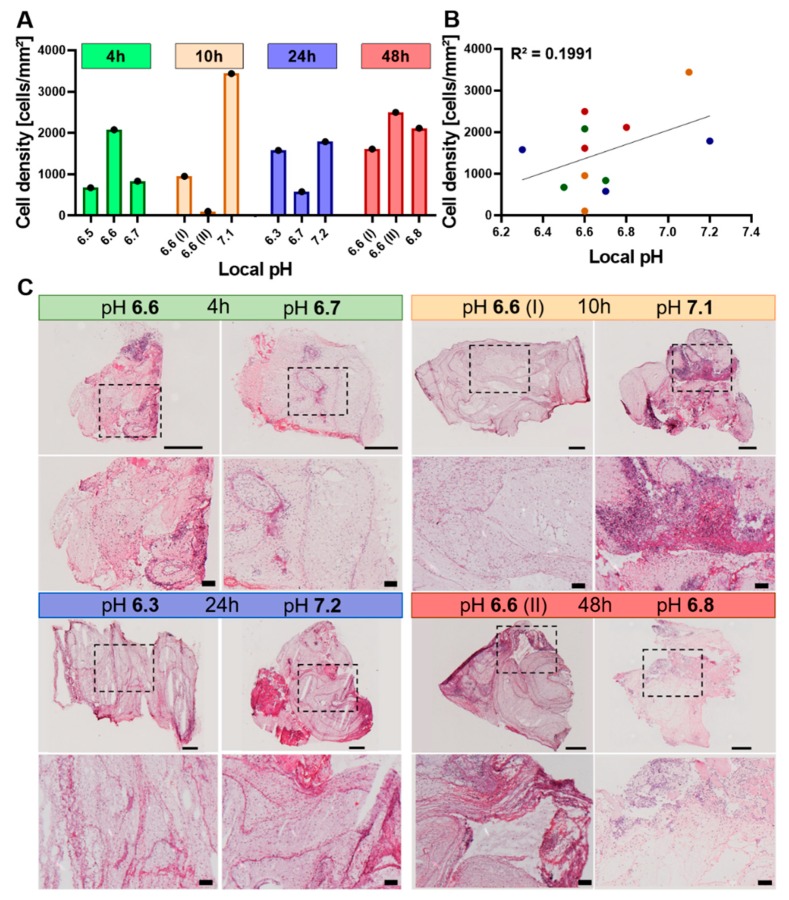
No direct link between the cellularity of hematoma and pH value. (**A**) Quantification of cells contained in the H&E-stained sections per mm^2^ of hematoma grouped by time point and pH (*n* = 3 for each time point). The total hematoma area was measured in mm^2^, cells were identified by hematoxylin staining the nuclei and the density was calculated by dividing the total cell count by the total hematoma surface area. (**B**) Cellular density of all hematomas (4, 10, 24, 48 h) plotted against local pH. Linear regression line is depicted for better visualization. Both parameters passed the D’Agostino–Pearson normality test (α= 0.05, QQ plot depicted in Supplementary Figure S2B,C). Hence, Pearson correlation was calculated, yielding an R^2^ value of 0.1991. (C) Representative H&E-stained sections of osteotomy hematomas at 4, 10, 24 and 48 h post osteotomy. Samples with a differing pH were chosen as examples for visualization. For each time point, the overview (top panel) and the close-up image (bottom panel) are shown in conjunction with the in vivo measured pH. The dashed box in the overview image indicates the area that is magnified in the bottom panel. Purple dots represent nuclei of cells, whereas the light to strong pink color stains structures such as cytoplasm, proteins and connective tissue. The scale bars indicate 500 µm for the overview image taken at 5x magnification or 100 µm for the close-up image taken at 10x magnification.

We next aimed to unravel the underlying cause for the inter-individual variability in pH values by quantifying the number of cells found in the osteotomy hematoma. As a result of injury-induced oxygen shortage, we hypothesized that the cells shift to anaerobic respiration and, thus, the number of cells would positively correlate with the amount of generated lactate, leading to a pH change. To test this hypothesis, the rat osteotomy hematomas were sectioned and H&E stained, and then the respective images were analyzed for cell number in conjunction with the measured pH (Figure 4). At all investigation time points, cellular density and local pH were not found to follow a similar pattern (Figure 4A), with cellular density not being correlated to pH (Figure 4B). At 10 h post injury, the hematoma with a pH of 7.1 shows areas of high cellular density that are not present in the more acidic hematoma (pH 6.6 (I)) as well as an overall higher cellular density (Figure 4A,C). At the 24 h time point, the cellular density of the most (pH 6.3) and least (pH 7.22) acidic hematoma exhibit approximately similar cellular densities (Figure 4A,C). Thus, this histological investigation disproved the hypothesis of a direct relationship between cell density and reduced pH.

#### 2.4.2. Differences in Metabolic Processes within the Hematoma Linked to Local pH

Since the cellularity of the hematoma did not correlate with the local pH value, we hypothesized that potential differences in metabolic processes within the cells of the hematoma could have caused different degrees of local acidification. Therefore, we investigated metabolites of the catabolic glucose pathway within the rat osteotomy hematomas. To avoid the influence of cellularity, the relative abundance of metabolites was quantified by normalization to the median intensity per sample using the whole data matrix of each hematoma. Based on the gene expression data in sheep, which showed a first upregulation of hypoxia-related genes at 12 h (Figure 2) and additional previous findings, indicating a major shift in cellular response in the time interval between 12 and 24 h [25], the fracture hematomas at 10 and 24 h were chosen for metabolomic analysis.

First, it was crucial to confirm the presence of D-glucose as the starting metabolite for all cellular respiratory processes. Calculating the ratios of relative quantities of D-glucose to the metabolites representing the first and the last step of the glycolytic cycle (D-glucose-6-phosphate and pyruvic acid) revealed that D-glucose was available in greater quantity than downstream metabolites for all the hematomas analyzed. This indicates that metabolic respiratory processes could occur and should not be stalled due to glucose shortage (Table 2).

**Figure 5:**
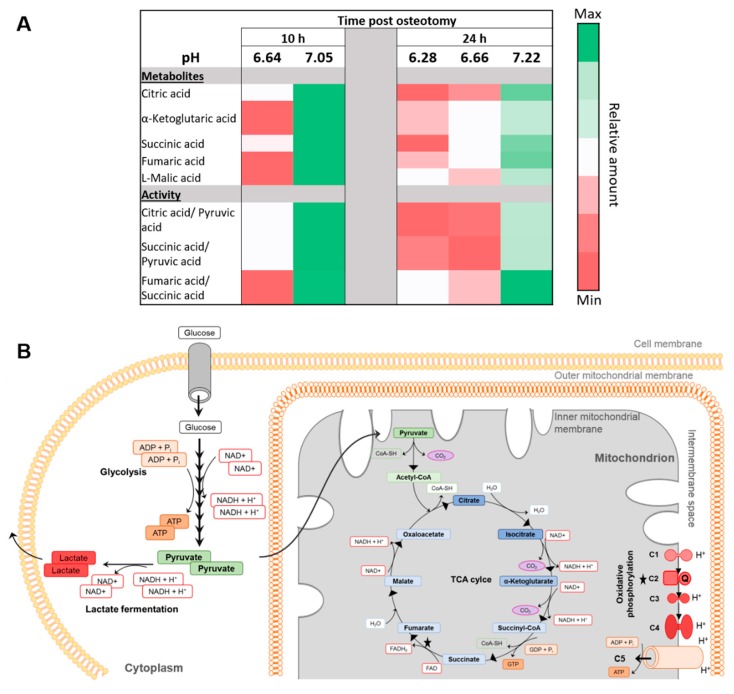
Metabolomic analysis of osteotomy hematomas—correlation between TCA activity and pH. (**A**) Heatmap of metabolites of the TCA and ratios between TCA and glycolysis products for 10 h (*n* = 2) or 24 h (*n* = 3) post osteotomy in conjunction with the individual pH measured in each hematoma samples that is represented here. (**B**) Simplified scheme of metabolic pathways underlying cellular respiration. Glycolysis occurs under aerobic and anaerobic conditions. Recovery of NAD^+^ under anaerobic conditions occurs in eukaryotic cells exclusively via lactate fermentation. Lactate is transported across cell membranes by cotransport with protons, thereby an acidification of the extracellular environment can occur. Under aerobic conditions, pyruvate enters the mitochondrion and participates in the TCA cycle. Energy in the form of ATP is generated during oxidative phosphorylation via proton shuttling through complex (**C**) 1, 3 and 4 into the intermembrane space and proton retrieval along the gradient by complex 5 (proton pump). Oxygen is needed at the end of the electron transport chain to generate H_2_O.

Under anaerobic conditions, lactic acid is produced and is pumped into the extracellular space, which results in an acidification of the local environment [33]. In our metabolomics analysis, lactic acid, which would give a direct indication for potential differences in anaerobic glucose catabolic pathways, could not be detected. However, differences in aerobic pathway activities could also provide evidence for a link of metabolic activity and pH. Thus, metabolites of the tricarboxylic acid (TCA) cycle as a key component of the aerobic respiration were studied. Figure 5A exhibits a metabolic heat map for relative quantities of metabolites belonging to the TCA cycle as well as for ratios of glycolysis and TCA metabolites in conjunction with the pH per hematoma at 10 and 24 h post injury. Figure 5B illustrates the pathways underlying cellular respiration, including the aerobic as well as the anaerobic energy generation via glucose catabolism. Interestingly, the relative quantity of TCA metabolites followed the same pattern as the pH—lower relative amounts of TCA metabolites were detected in hematomas with low pH compared to samples with higher pH of the same time point (Figure 5A). This was true for both the 10 and 24 h time points and is also reflected by the ratios of TCA metabolites against the end product of glycolysis, pyruvic acid. Moreover, forming a ratio of two consecutive TCA metabolites revealed the same trends. Taken together, this indicates that TCA activity seems to be higher in hematoma tissues with a higher pH. Thereby, aerobic metabolic activity can be related to local pH, with a higher pH being linked to higher activity in TCA activity, while local pH does not seem to be cellularity dependent.

## 3. Discussion

In this study, we investigated pH deviations from the physiological range during the very early to early phases post injury up to 48 h in vivo in sheep and rats to provide target pH values for novel pH-dependent treatment approaches. The pH changes were measured simultaneously in two injured tissues of the musculoskeletal system, the hematoma formed upon bone fracture and muscle trauma. To the best of our knowledge, this is the first study to examine pH changes in two musculoskeletal tissues in parallel in the same animal, thereby avoiding animal-specific variability. Local acidification was observed in the majority of both bone and muscle tissues in sheep, as well as in rats for which the mean pH dropped significantly below physiological pH (Figure 1, Figure 3). Gene expression studies in sheep fracture hematoma revealed the hypoxia-related upregulation of genes such as *GLUT1* to be initiated not immediately after injury, but starting at 4 h (Figure 2) [25]. Moreover, the hypoxia-sensitive expression of *GAPDH* could be demonstrated, which has been described for in vitro cell systems [29,30], but has not been previously reported for ex vivo sheep hematoma tissue. The observed local acidification supports published literature on pH deviations after fracture [24] and muscle injury [34]. However, in this study, we were focusing on very early to early healing phases, for which results of pH measurements were scarce and, so far, inconclusive. Moreover, we measured the extent of local acidification in vivo, which differentiates these findings from a number of previous studies that were summarized by Newman et al. [24]. Indeed, it became apparent during our study that the way the measurements are performed, e.g., the storage of the samples before measurement or the use of anti-coagulants, has a decisive influence on the results (Supplementary Figure S3), which indicates the significance of in vivo pH measurements. Interestingly, in vivo nuclear magnet resonance (NMR)-based pH measurements of rat fracture hematomas in another study showed the lowest pH of 7.19 within the fracture hematoma after two days, which was the first measurement time point [24]. In the current study, the average pH of the rat fracture hematoma at 48 h post fracture was 6.67 ± 0.1, the maximum pH measured at this time point was 6.77. Comparing all the previous studies mentioned [24], the results of the NMR and our pH measurements are among the most similar, despite utilizing different measurement methods. Of note, the non-invasive Raman spectroscopy approach utilized by Newman et al. [24] is an estimation of pH based on the magnetic resonances of phosphorus nuclei within tissues, which might explain the differences in the measured pH values. The local pH measured after muscle incision in another study [34] revealed a local mean acidification of 6.96 ± 0.04 at 24 h post incision, which is in strong agreement with our pH measurement in the muscle hematoma yielding mean pH values of 7.01 ± 0.18 at 24 h post osteotomy. Considering the acidification of the muscle or osteotomy hematoma intra-individually in sheep and rats, this study revealed a dissimilarity in extent and pattern of acidification among all animals. Thus, local acidification upon injury of these musculoskeletal tissues seems to occur without distinct tissue-dependent effects during the early healing phases, despite subtle differences in the kinetics of local recovery of physiological pH (Figure 3).

Moreover, we detected pronounced inter-individual differences in pH values of the hematomas at all investigated time points. Thus, we aimed to unravel potential underlying causes for this variability. The rupture of blood vessels upon injury creates hypoxic conditions (Figure 2) [2,3,4,35] and evokes the switch to anaerobic energy supply [3,4], resulting in lactate production (Figure 5B) and, thus, causing local tissue acidification (Figure 1, Figure 3) [23,24,36]. While histological analysis showed that cellular density within the hematoma is not causative for changes in tissue pH (Figure 4), metabolic analyses indicated that the pH is linked to the activity of cellular respiration pathways (Figure 5). In all samples, D-glucose was detectable, allowing for glycolysis to occur. According to the Pasteur effect, fermentation is inhibited in the presence of oxygen [37]. The metabolism of glucose is regulated by allosteric inhibition or activation of glycolytic enzymes. Whereas ATP and some metabolites of the glycolysis and TCA cycle decrease the rate of glycolysis, AMP, ADP and Pi (so-called low-energy metabolites) increase the rate [37,38]. Hypoxia leads to a decrease in oxidative phosphorylation (OXPHOS), which increases the abundance of low-energy metabolites and, thus, increases the glycolysis rate. To meet the energy demand, pyruvate has to be reduced to lactate instead of entering TCA cycling, thereby, a reduction in TCA activity links to an increase in lactate fermentation. Accordingly, when glycolysis coupled to lactate fermentation is the main pathway to ensure energy supply, aerobic respiration consisting of the TCA cycle and OXPHOS is reduced or stalled. TCA cycle and OXPHOS are coupled, since NADH and FADH2 need to be oxidized in the electron transport chain to allow for continuous TCA cycle activity. Since we could not detect lactate during our metabolomic analysis, as it is eluted at the beginning of the chromatogram and therefore not always detectable, we focused on metabolites of the TCA cycle. As mentioned above, the underlying assumption was that a higher activity in TCA cycle would reversely indicate a lower activity in anaerobic lactate fermentation, since ATP, NAD+, and FAD can be generated more effectively. Conversely, the reduction of TCA activity could be an indirect indication for increased lactate fermentation. It should be noted that additional processes affecting TCA metabolite abundance exist, as some TCA intermediates can be used for biosynthesis and be replenished by other mechanisms such as glutaminolysis [39]. However, this is beyond the scope of this investigation, which compares the hematomas of one tissue separately for each time point. According to our findings, in hematomas with a comparatively higher pH, the TCA cycle seems to be more active as shown by higher relative amounts of TCA metabolites and TCA metabolite to pyruvic acid ratios compared to hematoma samples of the same time point with a lower pH value. Remarkably, the ratio of fumaric acid over succinic acid also related to pH, i.e., higher pH values correspond to a higher ratio of these metabolites (Figure 5A). The enzyme succinate dehydrogenase subunit A catalyzes the conversion of succinic acid to fumaric acid. This enzyme participates in both the TCA cycle and the electron transport chain/OXPHOS and thereby couples both processes [39,40]. A higher rate of TCA cycle and succinate dehydrogenase activity could suggest that aerobic respiration can still take place and, therefore, less pyruvic acid would be fermented to lactate, leading to reduced tissue acidification and a less pronounced pH drop. Intriguingly, work by Khacho et al. suggests that mild extracellular acidosis, which can be evoked by hypoxia, can indeed induce a restructuring of mitochondria, thereby maintaining efficient ATP production by the mitochondria, despite oxygen shortage [33].

The knowledge gained in this study could pave the way for novel pH-mediated local treatments of musculoskeletal injuries by defining target values for pH sensitivity that would need to be matched by suitable biomaterial development. In a study by Chu et al., a nanosystem was developed that exhibits a more than doubled cumulative drug release in a pH 6.5 compared to a 7.4 environment [41], hinting at the technical feasibility of pH-mediated interventions after injury with mean target values of 6.69 and 6.89. The need for novel pharmacological interventions for musculoskeletal injuries becomes apparent by appreciating that 10%–20% of bone fracture cases exhibit delayed healing or develop into non-unions [42,43]. Injuries of the musculoskeletal system represent a major health issue, with fractures making up approximately 16% of injuries that were treated in medical care in the US in 2012 [44]. This percentage is predicted to rise due to an aging population with a prolonged life span [45]. An increase in age coincides with a rather pro-inflammatory systemic milieu, a phenomenon called “inflamm-aging” [46], and a higher prevalence of comorbidities such as obesity and diabetes, both limiting the regenerative capacity, e.g., in fracture cases [47]. Furthermore, as a clear relationship between the local pH of healing bone tissue and mineral deposition has been described [23], future studies could additionally investigate whether the extent of tissue acidification post injury corresponds to different healing capacities, thereby potentially further increasing the demand for such therapies. A prolonged local acidification phase can be assumed to impact the progression of bone healing, as abundant clinical evidence exists on systemic acidosis patients that present decreased bone quality and increased fracture risk [48,49,50,51,52,53] and mineral deposition occurs during the local alkalization phase that follows the initial acidification [23]. Some studies observed a direct link between the degree of systemic acidosis and fracture risk [51] or bone quality parameters such as bone mineral density (BMD) and bone loss rates [53]. Moreover, a pre-clinical study compared the pH dynamics and time to union as an indicator for mineral deposition in normal and experimentally delayed bone healing [36]. A prolonged phase of local acidification and overall reduced subsequent alkalization was detected for the delayed healing group that coincided with an increased time to union and, thus, slower mineral deposition. Together, these studies evidence a clear relation between systemic pH and bone quality as well as suggest a link between healing delay and hampered local pH dynamics.

In conclusion, despite high inter-individual differences, a significant pH drop was observed in our study within the rat osteotomy and muscle hematoma across all time points investigated. Further, the detected range of pH value changes in different tissues and individuals appears to be linked to the different TCA cycle activity within the injured tissue—even without a vascular supply of oxygen. Using biomaterials sufficiently sensitive to mild deviations from physiological pH, pH-mediated local treatments could be conceivable for both bone and muscle injuries. However, further studies are needed to identify a stable and reproducible pH drop across multiple tissues and injuries and to investigate a possible relationship between tissue acidification/alkalization dynamics and different healing outcomes in bone regeneration in clinically relevant animal models. For this, the comparison and longitudinal pH measurement in an age-related model of compromised healing [54] versus a young, normal healing model could prove insightful. Based on previous findings that provide evidence for an impaired re-vascularization in rodent fracture models in aged compared to young animals [32,55], a prolonged hypoxic phase causing an extended local acidification phase seems probable, but needs to be studied in depth. Since similar observations of age-dependent hampered angiogenic capacities were made in elderly human patients [56,57], a clinical application of such knowledge appears feasible, underlining the need for future research in this area.

## 4. Materials and Methods

### 4.1. Animal Studies

Depending on the expected size of the sample, specimen characteristics and measurement set-up, different pH sensors were employed within this study. For the initial pilot study in sheep, we focused on the development of local pH immediately post injury, while for the rat study, we monitored pH changes over a longer time frame to understand how local pH is affected by early hematoma maturation processes.

### 4.2. Sheep Study

The in vivo study in sheep was performed in accordance with the German Animal Welfare Act and was approved by the local animal protection authorities (Landesamt für Gesundheit und Soziales, LaGeSo; permit number G0216/13, approval: 28.10.2013; additional pH measurement approved in December 2015). The in vivo study was conducted in accordance with the German Animal Welfare Act, the National Institutes of Health Guide for Care and Use of Laboratory Animals and the Animal Research: Reporting of In vivo Experiments (ARRIVE) guidelines. The sheep in this trial were part of a larger study using adult female merino mix sheep (>2.5 years) for osteochondral healing, for which additional pH measurements at the endpoint of the study were approved by the local authorities, thereby following the “reduce” of the 3R principles (replace, reduce, refine) [27]. For the osteochondral healing study in which the pH measurement was included, the animals were held under deep anesthesia by a mixture of anesthesia gas (1.8%–2.0% isoflurane, 20%–30% nitrous oxide, pure oxygen) for 3 h before sacrifice. Within this time period, the pH measurement was integrated. The measuring period was determined by the above-mentioned ongoing animal study. Thus, only the immediate to early pH changes up to 3 h post injury could be investigated. Fentanyl dihydrogen citrate (Rotexmedica, Trittau, Germany) was given as intra-venous bolus injections repeatedly for analgesia.

A 6 mm drill hole in the shaft of the right tibia, as a representation of a forming hematoma after bone fracture, and/or a size-matched muscle trauma in the main body of the Vastus lateralis of the Musculus quadriceps by the use of a scalpel was created in a standardized manner without ceasing muscle function completely. Since the measurements occurred at the study end point and the forming hematoma was the tissue of interest, a complete osteotomy was avoided, as additional external fixation measures were not possible without disturbing the region used for the osteochondral study. The drill hole size and location were chosen to allow the entrance of the pH sensor into the medullary cavity. As the diameter of the bone marrow shaft is rather small, the proximal tibia was chosen. Here, the bone widens and allows immersion of the pH sensor in the bone marrow tissue. The pH sensor was inserted centrally into the defect, i.e., into the liquid phase of the injury-induced bleeding prior to coagulation, followed by suturing of the skin and pH measurement of the developing hematomas. The pH of the developing hematomas from both musculoskeletal tissues was measured. Measurements started right after injury and lasted for 180 min (max. measurement interval of 10 min) using a classical electrochemical pH microelectrode (8220BNWP Orion™ PerpHecT™ ROSS™ Thermo Fisher Scientific, Waltham, MA, USA). A total of 8 animals were measured, and the study size and allocation to the different hematoma groups were dictated by the lead study (4 animals: muscle hematoma; 4 animals: bone hematoma; and in 2 animals of both groups muscle and bone hematomas were created).

### 4.3. Gene Expression Analysis

Hematoma samples were generated in an earlier study and results from the expression analysis were partly published before in another context [25]. In brief, hematomas were generated by a stable fixated osteotomy in the tibia of sheep and harvested at 1, 4, 12, 24, 36, 48 and 60 h after surgery (*n* = 6 per time point). A total of 150 mg of hematoma tissue was used for total RNA isolation. A volume of 1 mL of TRIzol (Invitrogen, Carlsbad, CA, USA) was used for homogenization with an ultra turrax. Subsequently, 200 µl chloroform (VWR, Radnor, PA, USA) was applied, and samples were mixed and incubated for 10 min before centrifugation. The upper phase was collected and diluted in a 1:2 ratio with 2-propanol. After centrifugation, the pellet was resuspended in 75% ethanol. Following centrifugation, the pellet was dried at 37 °C and resuspended in 20 µl diethylpyrocarbonate (DEPC) water. After incubation with 80 µl DNase Mix (Quiagen, Düsseldorf, Germany) for 15 min, the procedure was repeated. The final pellet was resuspended in RNase-free water for 10 min at 50 °C. Quality and quantity of the RNA was measured by spectrophotometry, and RNA was stored at -80 °C until further use. cDNA synthesis was carried out using random primers (Invitrogen, Carlsbad, CA, USA) and MMLV-Reverse Transcriptase-RNaseH Minus (Promega, Madison, WI, USA). Primers were designed to perform at 62 °C and to give rise to 150–300 bp products (Tib Molbiol, Berlin, Germany). PCR was performed using the iQ^TM^Supermix (BIO-RAD, Hercules, CA, USA) and SYBR^®^Green (Invitrogen, Carlsbad, CA, USA) with an iQ^TM^5 Cycler (BIO-RAD, Hercules, CA, USA). Cyclophilin A was applied as a housekeeping gene after determining the best stability in expression within this tissue using geNorm. For data analysis, the 2^ΔCT^ method was used.

### 4.4. Rat Study

The in vivo study in rat was performed in accordance with the German Animal Welfare Act and was approved by the local animal protection authorities (Landesamt für Gesundheit und Soziales, LaGeSo; permit number G0017/16, approval: 31.03.2016). The study design is graphically illustrated in the Supplements (Figure S4). The in vivo study was conducted in accordance with the German Animal Welfare Act, the National Institutes of Health Guide for Care and Use of Laboratory Animals and the ARRIVE guidelines. In total, 24 female Sprague–Dawley rats (12 weeks old) were included in this study. They were kept under obligatory hygiene standards as monitored according the FELASA standards. Water and food were available to the animals ad libitum, and the rats were kept in groups and randomly assigned to the different investigation time points. The temperature was set to 20 ± 2 °C and a light/dark period of 12 h was utilized.

During the surgery, they all received a balanced isoflurane (Forene, Abott, Wiesbaden, Germany) anesthesia mixed with oxygen and analgesics. After induction of anesthesia, analgetic (Buprenorphine, RB Pharmaceuticals, Berkshire, UK) and antibiotic bolus (Clindamycine, Ratiopharm, Ulm, Germany) treatment and application of eye ointment, the animals were placed on a heating plate set at 37 °C. For the osteotomy, after exposure of the bone, an external fixator (RISystem, Davos, Switzerland) was mounted on the lateral aspect of the femur, an osteotomy gap of 5 mm was created with a Gigli saw and a saw guide, thereby allowing hematoma formation. In this study, in contrast to the sheep study, a complete osteotomy was required due to otherwise spatial limitation for the forming hematoma that could have impeded the pH measurements. For the muscle trauma, the skin at the shank was cut caudally, the Musculus gastrocnemius was mobilized bluntly and the underlying Musculus soleus was isolated and crushed 2 times for 20 s with a clamp.

To investigate the pH changes following injury in a pre-clinical model, measurements were conducted at 4, 10, 24 and 48 h post injury. Our studies showed [25,26] that hypoxic conditions and the cellular reactions stabilize between 4 and 12 h (upregulation of *GLUT1* and *HIF1α*), indicating that acidification due to anaerobic energy metabolism will increase during hematoma maturation. For the measurement in the small rat hematoma, a microinvasive needle-type optical pH microsensor (pH-1 micro combined with needle-type sensor (NTH-HP5), PreSens Precision Sensing, Regensburg, Germany) that includes a silica-based microfiber optic pH sensor system (sensor tip below 150 µm) for implementation in animal tissue [58] and on-the-spot continuous measurement within small volumes was utilized. This technique is based on the dual life time reference (DLR) method that uses two different fluorescent dyes, one dye being pH sensitive, and measures the pH-dependent phase shift of the two simultaneously excited fluorophores [59,60]. This microinvasive optical pH microsensor has been successfully utilized in previous studies to measure longitudinal pH in vivo in rodent animal models [34,61] and is moreover used for in vitro pH measurements [61,62]. Prior to the in vivo measurement, an in-house validation of the PreSens pH microsensors was accomplished by measuring the pH in human blood samples (Supplementary Figure S1A). For each investigation time point of the in vivo study, the pH within the hematomas was measured over a time period of two hours with a measurement interval of 10 s. For the time point 4 h after injury, the animals were kept in anesthesia until finalization. For all other time points, the animals recovered from anesthesia, were allowed normal activity and were treated with analgesia (Tramadolhydrochloride, Grünenthal, Aachen, Germany) that is optimized for this intervention for the other time points [63], before being anesthetized again for pH measurement. The last 30 min of the measurement were used for the evaluation of the pH values at the respective time points. The measurement was performed for *n* = 6 animals per time point (24 animals in total), and each measurement was an endpoint. Differences in final number of animals per time point depicted in the figures were due to sporadic premature deaths during anesthesia or technical issues with the optical pH sensor. After sacrifice, the osteotomy and muscle hematomas were harvested and stored at −80°C until further analyses.

### 4.5. Additional Analysis of Rat Fracture Hematoma

#### 4.5.1. Histological Analysis of Rat Fracture Hematoma

For the histological analysis, the fracture hematomas were cut into sections that were 5 µm thick using the CryoStat (CM3050 S, Leica, Wetzlar, Germany). After 10 minutes of fixation with 4% formaldehyde, hematoxylin and eosin (H&E) staining was applied. Images were taken at given magnifications (5x and 10x) using a bright field microscope (Axioskop 40, Zeiss, Oberkochen, Germany). Cell density was estimated by calculating the number of cells detectable due to the hematoxylin-stained nuclei per hematoma surface area in mm^2^ using the FiJi/ImageJ [64] software.

#### 4.5.2. Metabolomics Study of Rat fracture Hematoma

The sample preparation was performed according to metaSysX GmbH (Potsdam, Germany) standard procedure, a modified protocol from Giavalisco et al. [65]. Measurements were carried out with an Agilent Technologies Gas Chromatography (Agilent Technologies, Santa Clara, CA, USA) coupled to a Leco Pegasus HT mass spectrometer (Leco Instrumente, Mönchengladbach, Germany, which consists of an electron impact ionization source and a time of flight mass analyzer. Column: 30 m DB35; starting temp: 85 °C for 2 minutes; gradient: 15 °C per minute up to 360 °C. NetCDF files that were exported from the Leco Pegasus software were imported to “R”. The Bioconductor package TargetSearch was used to transform retention time to retention index (RI), to align the chromatograms, to extract the peaks, and to annotate them by comparing the spectra and the RI to the Fiehn Library and to a user-created library. Annotation of peaks was manually confirmed in Leco Pegasus. Analytes were quantified using a unique mass. The data was normalized to the sample median.

### 4.6. Statistics

Detailed information on all statistical analyses performed, including sample size and depicted values are included in the figure legends. A two-way Mann–Whitney U test was applied when indicated. To test for correlation, the D’Agostino–Pearson normality test was conducted (α= 0.05) on all parameters (local pH in osteotomy/muscle hematoma and cellular density on the entire data set including all time points), followed by Pearson correlation analysis and R^2^ calculation. To perform the statistical analysis, we used GraphPad Prism^®^ (Version 8.0; GraphPad Software, San Diego, CA, USA). The confidence interval was set to 0.95, and *p* < 0.05 was considered significant. Exact *p*-values for significant changes are stated in the figures.

## Figures and Tables

**Table 1:** Difference in pH values measured within the osteotomy and muscle hematoma per animal and time point. At all time points, the difference between both pH values was quite variable between animals and did not follow the same trend.

	∆ pH (Fracture Hematoma - Muscle Trauma)			
Time Point	4 h	10 h	24 h	48 h
**Individual ∆ pH per animal and time point**	0.53	−0.13	−0.33	−0.70
	0.92	−0.28	−0.16	−0.21
	−0.58	0.13	−0.71	−0.51
	−0.31	0.20	−0.66	0.01
	−0.78		0.38	
	−0.71			

**Table 2:** Ratios demonstrating glycolytic activity at 10 and 24 h post osteotomy.

Time Post Osteotomy	10 h		24 h		
Local pH	6.64	7.05	6.28	6.66	7.22
**D-glucose 6-phosphate/D-glucose**	0.001	0.003	0.171	0.004	0.017
**Pyruvic acid/D-glucose**	0.158	0.236	0.584	0.685	0.247

## Supplementary Materials

Supplementary materials can be found at https://www.mdpi.com/1422-0067/21/7/2513/s1.

## Abbreviations

DPEC	Diethylpyrocarbonate water
*GAPDH*	Gylcerinaldehyde-3-phosphate dehydrogenase
*GLUT1*	Glucose transporter 1
*HIF-1α*	Hypoxia-inducible factor-1α
*HMOX1*	Heme oxygenase-1
H&E	Hematoxylin and eosin
