# Supplementary material for: Early pH Changes in Musculoskeletal Tissues upon Injury—Aerobic Catabolic Pathway Activity Linked to Inter-Individual Differences in Local pH
Source: Int J Mol Sci. 2020 Apr 4;21(7):2513. doi: 10.3390/ijms21072513 (PMC7177603; doi:10.3390/ijms21072513)
Supplement: Supplementary file 1 [file ijms-21-02513-s001.pdf]

Supplements

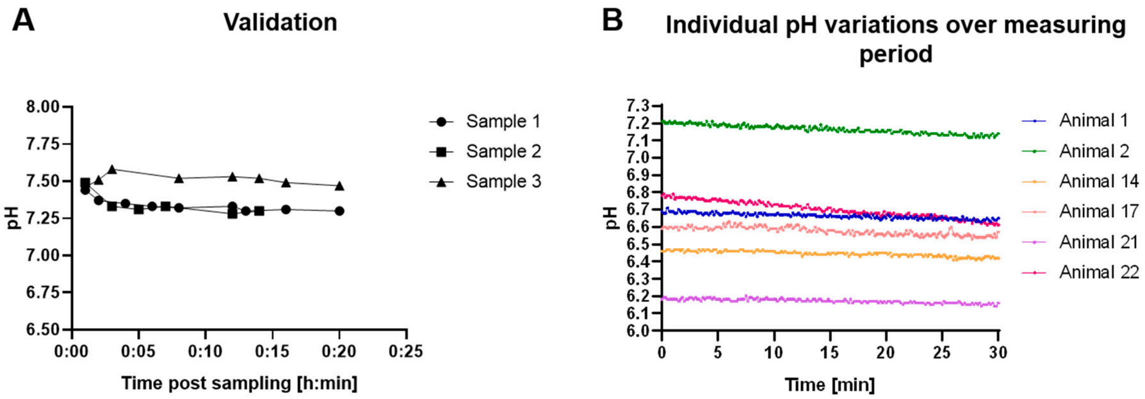

	Mean pH	SD	Overall mean pH	Overall SD
Sample 1	7.34	0.04	7.40	0.10
Sample 2	7.34	0.08		
Sample 3	7.51	0.04		

Animal	Mean	SD	Mean SD
1	6.66	0.02	±0.02
2	7.16	0.02	
14	6.45	0.02	
17	6.58	0.02	
21	6.17	0.01	
22	6.70	0.05	

Figure S1. Validation of the PreSens microsensor by measurement of human non-coagulated blood samples (n=3) **(A)** and by confirming the consistency of measurement in vivo in the rat osteotomy hematoma **(B)**. (A) measured mean pH of three human blood samples of 7.40 ± 0.1 is similar to the known physiological pH of human blood ranging from 7.35- 7.45. This validates the correct measurements by the pH sensor. **(B)** continuous recording (measurement interval: 10 s) over 30 min indicating a mean SD of individual pH per animal of ± 0.02.

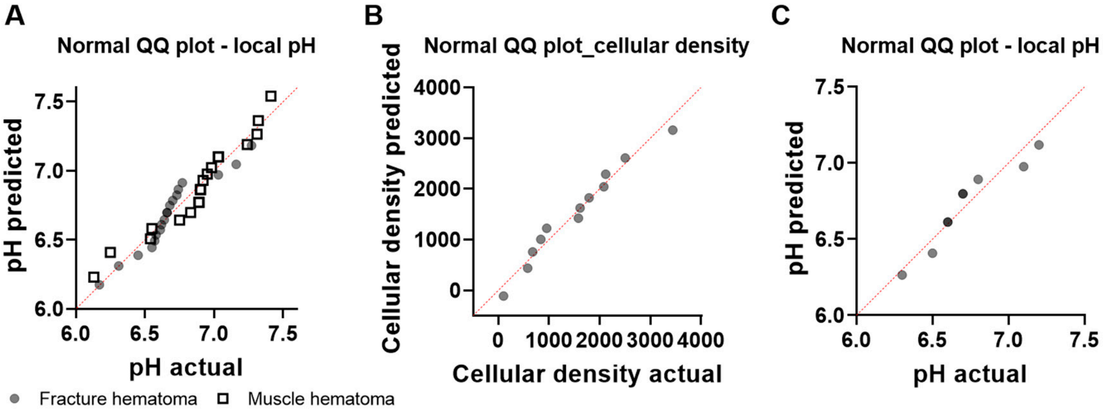

Figure S2. Normal QQ plots for visualization of normality analysis of data sets derived from the rat in vivo study. **(A)- (C):** Depicted is the fitting of all values belonging to the data set (actual values, x-axis) to the predicted values (y-axis), predicted under the assumption of normal distribution. **(A)** local pH of all fracture and muscle hematoma samples utilized to perform regression analysis of pH measured within both hematomas (Figure 3), n= 19. **(B)** local pH of fracture hematoma samples utilized for cellular density **(C)** calculation, for **(B)** and **(C)**: n= 12.

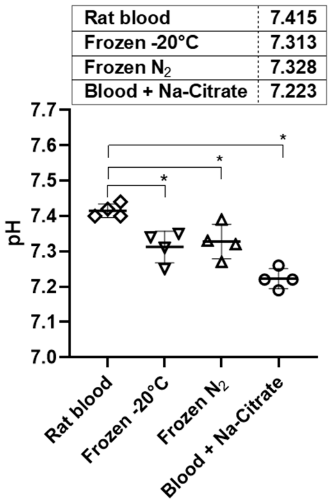

Figure S3. Storage and sample supplementation with anti-coagulant affect pH of sample (rat blood, n=4 independent blood samples from 4 rats). Direct pH measurement of rat blood, of previously frozen blood, either by freezing in -20°C environment or snap-freezing in liquid nitrogen and direct measurement in rat blood supplemented with the anti-coagulant sodium citrate. Two-way Mann-Whitney-U test was conducted to compare storage and supplementation with the directly measured pH.

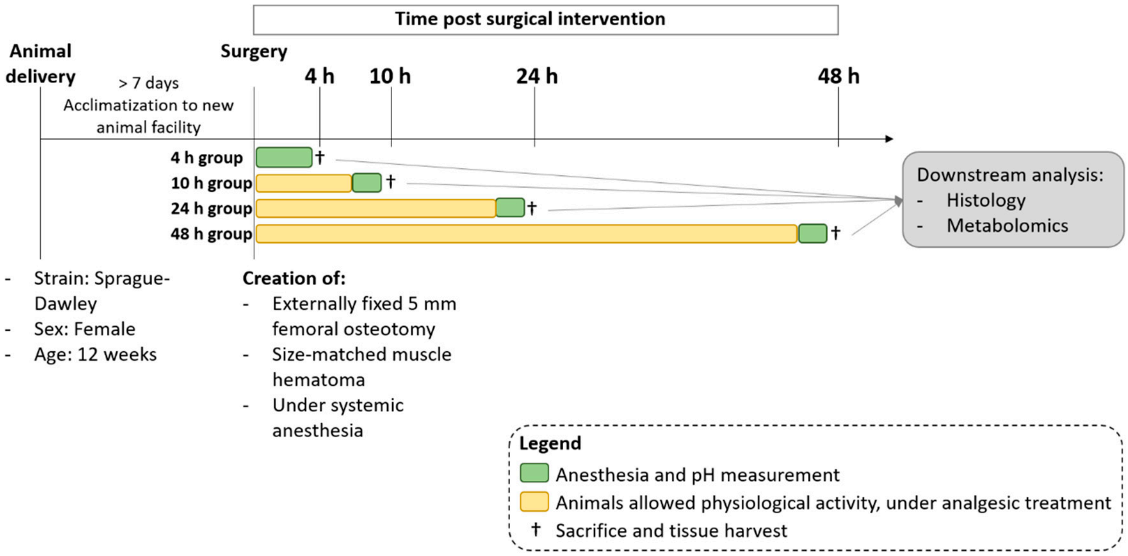

Figure S4. Study design of rat in vivo study.
